# The adult mortality profile by cause of death in 10 Latin American countries (2000–2016)

**DOI:** 10.26633/RPSP.2020.1

**Published:** 2020-01-14

**Authors:** Júlia Almeida Calazans, Bernardo Lanza Queiroz

**Affiliations:** 1 Universidade Federal de Minas Gerais Belo Horizonte Belo HorizonteMinas Gerais Brazil Universidade Federal de Minas Gerais, Belo Horizonte, Minas Gerais, Brazil.

**Keywords:** Cause of death, mortality, health transition, Latin America, Causas de muerte, mortalidad, transición de la salud, América Latina, Causas de morte, mortalidade, transição epidemiológica, América Latina

## Abstract

**Objective.:**

To investigate the adult mortality profile from eight causes of death in 10 Latin American countries (Argentina, Brazil, Chile, Colombia, Costa Rica, Ecuador, Mexico, Paraguay, Peru, and Uruguay) from 2000 to 2016.

**Methods.:**

The cause of death effect in adult mortality was calculated as the hypothetical gain in the average number of years lived in adulthood (15 to 60 years old), in a cause-deleted life table. Mortality information by cause, sex, and age group came from the World Health Organization.

**Results.:**

Although the adult mortality levels are very different among the 10 countries, the pattern of mortality by cause of death is very similar. All the countries are in the intermediate stages of the epidemiological transition, with chronic degenerative diseases being predominant. Among males, circulatory system diseases and external causes are the most important causes of death in terms of the average number of years lived in adulthood. Among females, the leading causes are circulatory system diseases and neoplasms.

**Conclusions.:**

Some studies have pointed out that Latin America exhibits severe difficulties in moving through some epidemiological transition phases, given the continuing high mortality from chronic diseases and violent deaths. However, between 2000 and 2016, there was a convergence among the 10 analyzed countries around the theoretical limit in the average number of years lived in adulthood. Countries that include Brazil, Colombia, Ecuador, Mexico, Paraguay, and Peru are still further away from this limit, but they have an enormous potential to increase the number of years lived in adulthood in the future.

## RESUMEN

**Objetivo.:**

Investigar el perfil de mortalidad en adultos por ocho causas de muerte en diez países latinoamericanos (Argentina, Brasil, Chile, Colombia, Costa Rica, Ecuador, México, Paraguay, Perú y Uruguay) del 2000 al 2016.

**Métodos.:**

Se calculó el efecto de la causa de muerte en la mortalidad en adultos como la ganancia hipotética en el número promedio de años vividos en la edad adulta (de 15 a 60 años de edad), mediante una tabla de vida con eliminación de causa. Los datos de mortalidad desglosados por causa, sexo y grupo etario proceden de la Organización Mundial de la Salud.

**Resultados.:**

Aunque los niveles de mortalidad en adultos son muy dispares entre los diez países, el patrón de mortalidad por causa de muerte es muy similar. Todos los países se encuentran en las etapas intermedias de la transición epidemiológica, en que las enfermedades degenerativas crónicas son predominantes. En los hombres, las enfermedades del aparato circulatorio y las causas externas son las causas más importantes en lo que respecta al número promedio de años vividos en la edad adulta. En las mujeres, las principales causas son las enfermedades del aparato circulatorio y las neoplasias.

**Conclusiones.:**

Algunos estudios han señalado que América Latina se enfrenta a graves dificultades para superar algunas de las fases de la transición epidemiológica, dada la mortalidad elevada continua por enfermedades crónicas y muertes violentas. Sin embargo, entre los años 2000 y 2016 hubo una convergencia entre los diez países analizados en torno al límite teórico en el número promedio de años vividos en la edad adulta. Algunos países, como Brasil, Colombia, Ecuador, México, Paraguay y Perú, aún se encuentran lejos de ese límite, si bien tienen un gran potencial para aumentar el número de años vividos en la edad adulta en el futuro.

## RESUMO

**Objetivo.:**

Investigar o perfil de mortalidade de adultos por oito causas de morte em 10 países da América Latina (Argentina, Brasil, Chile, Colômbia, Costa Rica, Equador, México, Paraguai, Peru e Uruguai) no período de 2000 a 2016.

**Métodos.:**

O efeito das causas de morte sobre a mortalidade de adultos foi calculado como ganho hipotético no número médio de anos vividos na idade adulta (15 a 60 anos), utilizando uma tabela de vida com eliminação de causa. As informações de mortalidade por causa, sexo e faixa etária foram obtidas com a Organização Mundial da Saúde.

**Resultados.:**

Embora os níveis de mortalidade em adultos sejam muito diferentes entre os 10 países, o padrão de mortalidade por causa de morte é muito semelhante. Todos os países estão nas etapas intermediárias da transição epidemiológica, com predominância de doenças crônico-degenerativas. Entre os homens, as doenças do sistema circulatório e as causas externas são as causas de morte mais importantes em termos do número médio de anos vividos na idade adulta. Entre as mulheres, as principais causas são as doenças do sistema circulatório e neoplasias.

**Conclusões.:**

Alguns estudos indicam que a América Latina apresenta sérias dificuldades em passar por algumas fases da transição epidemiológica, dada a manutenção de uma alta mortalidade por doenças crônicas e mortes violentas. No entanto, entre 2000 e 2016, houve uma convergência entre os 10 países analisados em torno do limite teórico do número médio de anos vividos na vida adulta. Países como Brasil, Colômbia, Equador, México, Paraguai e Peru ainda estão mais longe desse limite, mas têm um enorme potencial para aumentar o número de anos vividos na vida adulta no futuro.

Most Latin American countries have experienced an accelerated decrease in mortality since the mid-1950s, translating into continuous improvements in life expectancy. Although this phenomenon stems from several factors, the reductions in infant mortality, especially due to the decline in infectious and parasitic diseases, explain most of the advances (1-4).

The idea that the reduction in infant mortality is the only cause of this process does not tell the complete story. Reductions in adult mortality are relatively small compared to infant mortality, but that does not mean they do not contribute to overall changes in mortality. Soares (2) shows that the probability of an individual dying between ages 15 and 60 years old in Latin America fell around 35% from 1960 to 2000. As Latin America moves forward in the demographic transition and the epidemiological transition, mortality declines are expected to depend less on young ages and more on adult and advanced ages (5-8).

These reductions in mortality for persons aged 15 to 60 are associated with changes in the profile of the leading causes of death (3, 9-11). During most of the transition process, the reduction in infectious diseases helped to explain the decline in adult mortality. In recent decades, however, the role played by changes in heart diseases, neoplasms, diabetes, and other chronic degenerative diseases has become more prominent (2-4, 9-11). Socioeconomic changes and adoption of deleterious behaviors, such as poor diet, sedentarism, and smoking, have been the leading factors for the increase in mortality from chronic degenerative diseases in Latin America in recent years (3, 4, 12, 13).

Another striking feature of the mortality pattern in Latin America, especially in Brazil, Colombia, Ecuador, and Mexico, has been the increasing number of deaths from violence and accidents since the 1980s. These levels are still high in many countries, and there is a concentration of these deaths among young adult males (3, 11, 13-18). Frenk et al. (18) argue that the mortality from these causes in Latin American countries is independent of the epidemiological transition and is strongly associated with political instability, economic inequality, social segregation, and drug trafficking.

The pace of change in the mortality pattern is very heterogeneous (3, 4, 5, 18). The diversity in behavior patterns, development levels, and technology assimilation processes among and within countries has a fundamental role in determining the decline in mortality levels, as well as the changes in the pattern of causes of death across Latin America.

The epidemiological profile changes in Latin America have been widely studied in recent decades (2-6, 8, 11, 13, 17, 19-26). However, to our knowledge, there are few studies performing cross-country comparisons and discussing regional similarities and differences in the process of epidemiological transition (2-5, 11, 17). Further, these studies are not directly comparable since they have used different countries, periods, and mortality indicators in their analyses. However, some indications about the epidemiological transition in the region can be elucidated from this extensive literature.

Frenk et al. (11) analyze the mortality profile by causes of death in Latin American and Caribbean countries in the 1980s and suggest that the countries were experiencing a different epidemiological transition model from developed countries, since it was possible to observe simultaneously a high incidence of death causes characteristic of pre- and posttransition stages (extended polarized model).

On the other hand, Soares (2), Palloni and Pinto (3), and Marinho et al. (4) summarize the contribution that reductions in some causes of deaths make to gains in life expectancy between 1950 and 2000, with a more considerable advance in the epidemiological transition in several Latin American and Caribbean countries. More recently, Dávila-Cervantes and Agudelo-Botero (17) have analyzed the contribution of avoidable deaths to the change in life expectancy in Argentina, Chile, Colombia, and Mexico, and Alvarez et al. (5) have investigated how the changes in mortality by causes of death can impact the life-span variation in a series of Latin American and Caribbean countries. These two studies conclude that avoidable deaths continue to have a major impact on mortality, but with a great disparity among countries. In addition, Alvarez et al. (5) point out that the dispersion of avoidable deaths through the age span makes the most substantial contribution to the gap between the Latin American and Caribbean countries and developed countries.

The objective of this study is to examine the mortality profile of adults (15 to 60 years old) by cause of death in 10 Latin American countries from 2000 to 2016. The effect of each cause of death will be calculated as the hypothetical gain in the average number of years lived in adulthood, using cause-deleted life tables. Thus, this paper seeks both to broaden the understanding of the adult mortality profile and its recent changes and to provide evidence that may contribute to the discussion about the process of increasing longevity in these countries.

## MATERIALS AND METHODS

This study followed a longitudinal ecological design that analyzed the temporal evolution of mortality by cause of death in the average number of years lived in adulthood at the national level.

### Data

Mortality data quality in the Latin America has significantly improved in recent decades. However, the incompleteness of death counts coverage is still a problem. Therefore, age-specific mortality rates from the World Health Organization (WHO) life tables (27) were used to calculate the average number of years lived in adulthood since those rates are already corrected by the level of completeness of death counts registration. The number of deaths by cause, sex, and age group also comes from the WHO (28). Missing information on age and sex were distributed according to the observed structure for the deaths with declared age and sex.

The 10 countries selected for analysis were Argentina, Brazil, Chile, Colombia, Costa Rica, Ecuador, Mexico, Paraguay, Peru, and Uruguay. The choice of countries was made qualitatively, ensuring that the selected set had countries with different socio-economic conditions and different mortality levels. In addition, these countries together have approximately 83% of the population of Latin America (29). The data on the number of deaths by cause was not available for Colombia (2016), Costa Rica (2015 and 2016), Peru (2016), and Uruguay (2011).

The causes of deaths were classified according the International Classification of Diseases (ICD-10). The causes analyzed were diabetes (E10-E14), respiratory system diseases (J00-J99), circulatory system diseases (I00-I99), neoplasms (C00-D48), external causes (V01-Y98), HIV/AIDS (B20-B24), other infectious and parasitic diseases (A00-B99), and other causes that are not included in these first seven groups. This selection took into account their relevance within the theoretical framework of the epidemiological transition proposed by Omran (9). In addition, these causes represented around 65% of the adult deaths in the analyzed countries between 2000 and 2016 (27).

Also, the study assumes that the age at entry in adulthood could be set at 15. At this age, low mortality levels in childhood are replaced by increasing risks seen in young adults. Finally, the study covers an important age range, up to age 60, but avoids problems that are common in working with old-age mortality.

### The average number of years lived in adulthood

Adult mortality was investigated in terms of the average number of years lived in adulthood, as proposed by Hoem (30). Very similar to the concept of life expectancy, this indicator can be interpreted as the average number of years that each individual expects to live from age 15 to age 60 if he or she has experienced the prevailing mortality rates during the period. This indicator can be expressed by the ratio between two life table functions, ${}_{45}e_{15}^{0}=\frac{{}_{45}L_{15}}{l_{15}}$, where ${\;}_{45}L_{15}$ represents the number of person-years lived between the ages 15 and 60 (${\;}_{45}L_{15}$) and $l_{15}$ represents the number of people living at age 15.

Contrasting the life expectancy at age x, the average number of years lived in adulthood has a maximum value. If there is no death in a population between the ages of 15 and 60, the value of this indicator will necessarily equal 45 years. It is useful to both analyze countries over time comparatively and to examine how close to or distant from this limit they are.

### Cause-deleted life tables analysis

The effect of each cause of death on total mortality was calculated as the hypothetical gain in mortality when the mortality rates of a particular cause of death are arbitrarily set to equal zero and the mortality force for all other causes remains constant, using a cause-deleted life table. Therefore, all life table functions are recalculated considering this hypothetical suppression of each cause of death. In addition, the effect of each cause of death on the average number of years lived in adulthood can be calculated based on these new functions. The cause-deleted life table results were presented in terms of percentage gains in the average number of years lived in adulthood, making it possible to compare different mortality levels among countries and over time.

The results from a cause-deleted life table should be viewed as a counterfactual exercise and not an epidemiological forecast. Although the complete elimination of a cause of death is unlikely in current medical circumstances, this type of analysis may be useful for planning, prevention, and disease management (31).

## RESULTS

### Adult mortality in Latin American countries

**FIGURE 1.**
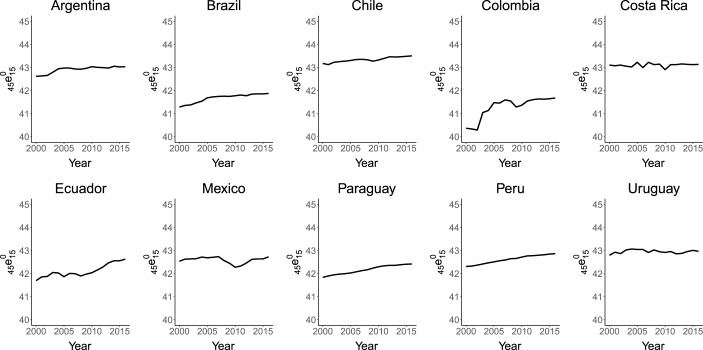
The average number of years lived in adulthood in 10 Latin American countries, males, 2000 to 2016

***Source:*** World Health Organization (27).

**FIGURA 2.**
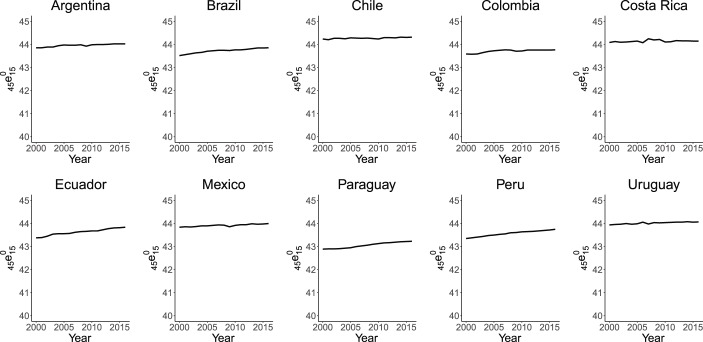
The average number of years lived in adulthood in 10 Latin American countries, females, 2000 to 2016

***Source:*** World Health Organization (27).

Figure 1 (males) and Figure 2 (females) show the average number of years lived in adulthood from 2000 to 2016. Costa Rica and Chile present the highest average number of years lived in adulthood, while Colombia has the lowest average number of years among males, and Paraguay has the lowest average number of years among females. All countries have experienced an increase in adult longevity in the period analyzed, converging more and more towards the theoretical limit of the average number of years lived in adulthood (45 years). In 2000, the difference between the theoretical limit and the highest observed average number of years lived in adulthood, in Chile, was 1.82 years among males and 0.79 years among females. In 2016, the difference was only 1.49 years among males and 0.68 years among females.

The improvements over the period varied among the 10 countries. Costa Rica, Chile, and Uruguay had small increases in the average number of years lived compared to the other analyzed countries. Colombia had the fastest growth rate in adult survival among males, with the average number of years lived in adulthood increasing 1.31 years between 2000 and 2016. Ecuador had the largest improvement for females, with an increase of 0.46 years in the same period.

The increase in the average number of years lived in adulthood in Colombia was remarkable, especially among males. During the last four decades of the twentieth century, political instability, armed conflict, and drug trafficking resulted in the highest homicide rates in the world (15, 34). Poverty and the precariousness of health services also directly affected mortality. Since 2002, with political stability, the country has been substantially improving its social indicators and reducing its mortality levels.

On the other hand, the average number of years lived in adulthood decreased continuously for males and females in Mexico between 2007 and 2010. Part of the increase in adult mortality rates in Mexico can be explained by the rise in violence and the mortality from chronic diseases, such as heart disease and diabetes (12, 13, 16), which will be discussed later in this article. Since 2010, the average number of years lived in adulthood has risen again in Mexico, especially due to public policies aimed at the prevention and treatment of chronic diseases.

Differences between countries have been declining over time. In 2000, the difference between the highest average number of years lived in adulthood and the lowest average number of years lived was 2.81 years for males and 1.34 years for females. By 2016, this difference was 1.84 years for males and 1.08 years for females.

### Estimates of adult mortality by causes of death in Latin American countries

**FIGURA 3.**
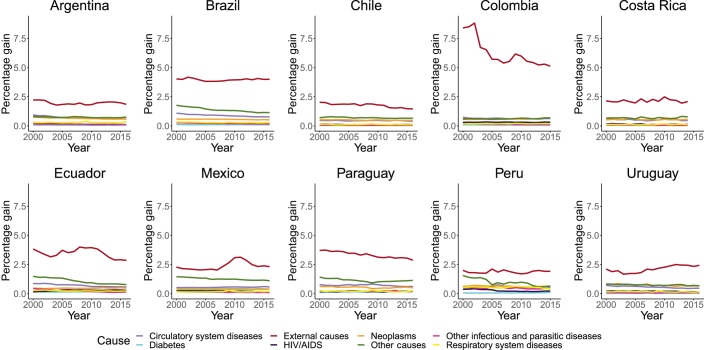
Percentage gain in the average number of years lived between the ages of 15 and 60 years old, if each of the causes of death is hypothetically deleted, in 10 Latin American countries, males, 2000 to 2016^a^

***Source:*** World Health Organization (27, 28).

^a^ Data on the number of deaths by cause is not available for Peru (2016), Colombia (2016), Costa Rica (2015 and 2016), and Uruguay (2011). For the construction of the chart, information from Uruguay (2011) was calculated from linear interpolation between the years 2010 and 2012.

**FIGURA 4.**
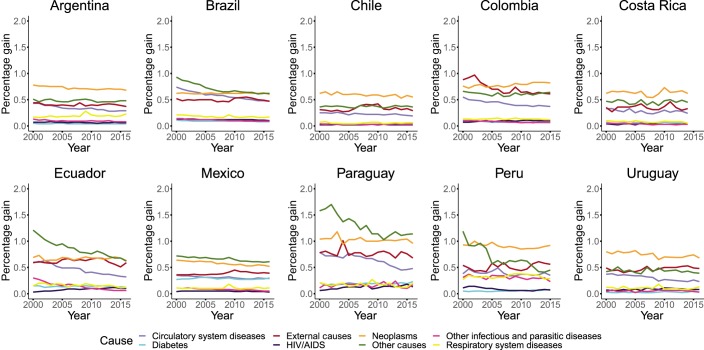
Percentage gain in the average number of years lived between the ages of 15 and 60 years old, if each of the causes of death is hypothetically deleted, in 10 Latin American countries, females (2000 to 2016)^a^

***Source:*** World Health Organization (27, 28).

^a^ Data on the number of deaths by cause is not available for Peru (2016), Colombia (2016), Costa Rica (2015 and 2016), and Uruguay (2011). For the construction of the chart, information from Uruguay (2011) was calculated from linear interpolation between the years 2010 and 2012.

This section presents the percentage gains in the average number of years lived in adulthood if a specific cause of death had been hypothetically suppressed, based on cause-deleted life tables for males (Figure 3) and for females (Figure 4).

With the theoretical elimination of circulatory system diseases, Argentina and Brazil would have had the largest gains in the average number of years lived in adulthood among males, and Brazil and Paraguay would have had the largest gains among females. That is, the effect of these diseases in the adult mortality level is higher in these three countries than in the other investigated countries. Between 2000 and 2016, there was a reduction in the impact of circulatory diseases in adult mortality. The largest reductions were in Argentina, Brazil, and Ecuador for males and in Brazil, Ecuador, and Paraguay for females. Mexico is the only country in which there was increased participation of these diseases for adult males over time.

With the theoretical elimination of neoplasms, Argentina and Uruguay would have had the largest gains in the average number of years lived in adulthood among males, and Paraguay and Peru would have had the largest gains among females. Costa Rica and Chile would have had the smallest gains. Neoplasm trends vary significantly among countries. Between 2000 and 2016, Colombia, Costa Rica, Ecuador, and Peru would have had an increase in the gains in the average number of years lived in adulthood among males with a hypothetical elimination of neoplasm mortality, while the other countries would have shown a decrease in the gains. Only Colombia and Costa Rica would have had an increase in the gains in the average number of years lived in adulthood among females.

The effect of diabetes mellitus on the average number of years lived in adulthood is small as compared to the effect of circulatory diseases and neoplasms. However, one point that deserves to be highlighted is the large differential in diabetes mortality. If the diabetes mortality rates were arbitrarily zero, the gains in the average number of years lived in adulthood in Mexico would be twice as large as the gains in other countries among males, and 1.3 times as large as the gains in other countries among females. Chile and Uruguay would have had the lowest survival gains if diabetes had been suppressed for both sexes. Between 2000 and 2016, the diabetes effect on the average number of years lived in adulthood increased in Mexico, Paraguay, Peru, and Uruguay. For other countries, this effect remained stable or dropped slightly.

In the case of respiratory diseases, Peru would observe the largest gains in adult survivorship, while Chile and Costa Rica would have the smallest gains. Between 2000 and 2016, Argentina, Colombia, Mexico, and Paraguay showed an increase in the participation of these causes among males; Argentina, Peru, and Uruguay presented an increase among females.

External causes also play an important role in adult mortality in Latin America, becoming the cause that kills more adult men in the region. Brazil, Colombia, Ecuador, Paraguay, and Mexico would have had the largest gains in survival if these causes had been suppressed in a cause-deleted life table. The effect of these causes in Colombia in 2000 was very high because of the armed conflict. From 2000 to 2016, only Mexico, Peru, and Uruguay would have had an increase in the gains in the average number of years lived in adulthood if external causes had been suppressed. The reduction of the impact of external causes would have been more pronounced for Colombia, Ecuador, and Paraguay than for the other countries.

It is worth noting that the effects of external causes on male mortality are dramatically higher than the effects on female mortality. In Brazil and Colombia, for example, the gains in the average number of years lived in adulthood would be seven times higher among men than among women.

The effect of HIV/AIDS and other infectious and parasitic diseases on adult mortality is substantially lower than the effect of other causes of deaths in Latin America. Brazil would present the largest gains in the number of years lived in adulthood in suppressing HIV/AIDS for both sexes, while Ecuador and Peru would present the largest gains in the number of years lived in adulthood in suppressing the other infectious diseases. For both HIV/AIDS and other infectious diseases, Chile would have the smallest gains in the average number of years lived in adulthood.

HIV/AIDS and other infectious diseases present more fluctuations over time than do chronic degenerative diseases. However, it is possible to see a general trend for adult mortality. Between 2000 and 2016, only Colombia, Ecuador, and Paraguay would have experienced an increase in the gains in the average number of years lived in adulthood when HIV/AIDS mortality rates were arbitrarily zero. For other infectious diseases, it is possible to observe a reduction in the gains in the average number of years lived in adulthood for all the investigated countries from 2000 to 2016.

## DISCUSSION

Despite the considerable heterogeneity in adult mortality in Latin America, the countries analyzed in this study can be divided into two groups (1, 2, 3, 4). One group comprise the precursor countries in the process of demographic transition, such as Argentina, Chile, Costa Rica, and Uruguay, which present the highest average number of years lived in adulthood. The second group are the trailing countries, such as Brazil, Colombia, Ecuador, Mexico, Paraguay, and Peru, with the lowest average number of years lived in adulthood.

Although adult mortality levels are very different among the countries, the pattern of mortality by cause of death is very similar. All the countries are in the intermediate stages of the epidemiological transition, with chronic degenerative diseases being predominant. Among males, circulatory system diseases and external causes are the most important causes of death in terms of the average number of years lived in adulthood. Among females, the leading causes are the circulatory system diseases and neoplasms.

Palloni and Pinto-Aguirre (3) observed that adult mortality from chronic degenerative diseases was beginning to fall in the precursor countries, especially in Chile and Costa Rica, but it continued to rise in the trailing countries between 1950 and 2000. With the exception of Mexico, in all our study countries mortality from circulatory diseases declined between 2000 and 2016. In addition, the precursor countries already showed a marked reduction in the contribution of neoplasms to adult mortality.

The impact of diabetes mortality is relatively lower than the impact of the other chronic degenerative causes mentioned here. However, its relevance to adult mortality in Mexico is noteworthy, given that it is significantly larger than in other countries. This finding corroborates other studies that discuss the effect of diabetes on the evolution of life expectancy in Mexico (13, 16).

One of the explanations for the predominance of chronic degenerative diseases in adult mortality is the population aging due to the demographic transition taking place in the Latin American countries since the 1970s (34). However, the transformation in the epidemiological profile goes beyond the change in the age profile of the population. It also includes important changes in adult mortality in terms of the average number of years lived in adulthood. This indicator is not influenced by age structure (30). Several studies have provided evidence that socioeconomic conditions and lifestyle changes have been important drivers of adult mortality trends in Latin America in recent years (2, 3, 4, 12, 13).

Primary health care services have expanded in some Latin American countries in recent years. However, health service preventive care for individuals with chronic conditions is still a challenge due to fragmentation, low efficiency, and limited response capacity (18, 32, 33). This discussion is even more germane in Brazil, where circulatory system diseases have a more significant impact than in other countries.

Another critical obstacle to reducing adult mortality in Latin American countries is external causes. Poverty, social inequality, economic instability, and drug trafficking sustain high levels of violent mortality (14, 15, 18, 35). In recent decades, various endeavors have helped reduce poverty and alleviate social inequalities in Latin America. These efforts have included social education policies, conditioned income transfer policies such as Oportunidades in Mexico and Bolsa Família in Brazil, and the strengthening of national labor markets. However, the adoption of multisectoral policies that consider violent mortality simultaneously as a social and public health problem is still a challenge for all the countries in the region (33, 35).

The study has some limitations that need to be acknowledged. The first limitation concerns the quality of mortality data for all Latin American countries. Age-specific mortality rates from WHO life tables are already adjusted for death records coverage, but there may still be a problem in classifying causes of death. Therefore, to minimize this problem, the analysis focused on the most recent periods. The second limitation concerns the generalization of results for the region as a whole. Although our country selection sought to ensure social, demographic, and economic heterogeneity, any generalization can be biased and must be done carefully. Finally, cause-deleted life tables are a hypothetical analysis of what would happen to mortality if a particular cause was eliminated. Nevertheless, they are an important approach for understanding the epidemiological transition in the region.

## Conclusion

The study highlights the changes in mortality by causes of death in a series of Latin American countries and how these changes might impact the evolution of the average number of years lived in adulthood. Some studies have pointed out that Latin America presents severe difficulties in moving through some phases of this transition, given the continuing high rates of violent deaths and the high mortality rates from chronic diseases. Between 2000 and 2016, there was a convergence among the 10 countries analyzed here around the theoretical limit of the average number of years lived in adulthood. However, the trailing countries are still further away from this limit, but with an enormous potential to increase the years lived in adulthood in the future.

## Author contributions.

Both authors participated in the conception of the study, estimation and interpretation of the results, writing of the manuscript, and critical revision of the manuscript regarding the important intellectual content until the approval of the final version.

## Acknowledgments.

The authors would like to thank the Brazilian Science Foundation (Conselho Nacional de Desenvolvimento Científico e Tecnológico – CNPq, Projeto Edital Universal 421183/2018-7), as well as the reviewers for their comments.

## Disclaimer.

The authors hold sole responsibility for the views expressed in the manuscript, which may not necessarily reflect the opinion or policy of the RPSP/PAJPH and/or PAHO.

## References

[1] 1. Arriaga E, Davis K. The pattern of mortality change in Latin America. Demography. 1969 Aug;6(3):223–42 10.2307/2060393 21331845

[2] 2. Soares R. On the determinants of mortality reductions in the developing world. Popul Dev Rev. 2007 Jun;33(2):247–87.

[3] 3. Palloni A, Pinto-Aguirre G. Adult mortality in Latin America and the Caribbean. In: Rogers RG, Crimmins EM, eds. International handbook of adult mortality. Dordrecht, Netherlands: Springer; 2011:101–32.

[4] 4. Marinho F, Soliz P, Gawryszewski V, Gerger A. Epidemiological transition in the Americas: changes and inequalities. Lancet. 2013 Jun;381(17–19):S89. doi: 10.1016/S0140-6736(13)61343-4

[5] 5. Alvarez JA, Aburto JM, Canudas-Romo V. Latin American convergence and divergence towards the mortality profiles of developed countries. Popul Stud. 2019 Jun:1–18. doi: 10.1080/00324728.2019.1614651 10.1080/00324728.2019.1614651 31179848

[6] 6. Gonzaga MR, Queiroz BL, De Lima EE. Compression of mortality: the evolution in the variability in the age of death in Latin America. Rev Latinoam Poblac. 2018 Aug:9–35. doi: 10.31219/osf.io/pdnfk

[7] 7. Wilmoth JR. Demography of longevity: past, present, and future trends. Exp Gerontol. 2000 Dec;35(9–10):1111–29. doi: 10.1016/S0531-5565(00)00194-7 10.1016/s0531-5565(00)00194-7 11113596

[8] 8. Solís P, García-Guerrero V. ¿Caminos divergentes a la baja mortalidad? El incremento en la esperanza de vida y la desigualdad de años vividos en América Latina y Europa. Estud Demogr Urbanos Col Mex. 2019 May/Aug;34(2):365–93. doi: 10.24201/edu.v34i2.1796

[9] 9. Omran A. The epidemiologic transition: a theory of the epidemiology of population change. Milbank Q. 1971;49(4):509–38. 5155251

[10] 10. Horiuchi S. Epidemiological transitions in human history. In: United Nations, Population Division. Health and mortality: issues of global concern. New York: UN;1999:54–71.

[11] 11. Frenk J, Frejka T, Bobadilla JL, Stern C, Lozano R, Sepúlveda J, et al. La transición epidemiológica en América Latina. Bol Oficina Sanit Panam. 1991;111(6):485–96. 1838685

[12] 12. Beltrán-Sánchez H, Thomas D, Teruel G, Wheaton F, Crimmins EM. Links between socio-economic circumstances and changes in smoking behavior in the Mexican population: 2002–2010. J Cross Cult Gerontol. 2013 Sep;28(3):339–58. 10.1007/s10823-013-9203-8 PMC3777536 23888371

[13] 13. Canudas-Romo V, García-Guerrero V, Echarri-Cánovas C. The stagnation of the Mexican male life expectancy in the first decade of the 21st century: the impact of homicides and diabetes mellitus. J Epidemiol Community Health. 2015;69(1):28–34. doi: 10.1136/jech-2014-204237 10.1136/jech-2014-204237 25252678

[14] 14. Canudas-Romo V, Aburto JM. Youth lost to homicides: disparities in survival in Latin America and the Caribbean. BMJ Glob Health. 2019;4(2):e001275. doi: 10.1136/bmjgh-2018-001275 10.1136/bmjgh-2018-001275 PMC6509612 31139444

[15] 15. Dávila CA, Pardo-Montaño AM. Factores socioeconómicos asociados con la mortalidad por homicidios en Colombia, 2000–2014. Cien Saude Colet. 2019 Aug;24(8):2793–804. doi: 10.1590/1413-81232018248.29142017 10.1590/1413-81232018248.29142017 31389528

[16] 16. Canudas-Romo V, Aburto JM, García-Guerrero VM, Beltrán-Sánchez H. Mexico’s epidemic of violence and its public health significance on average length of life. J Epidemiol Community Health. 2017;71(2):188–93. 10.1136/jech-2015-207015 PMC5284477 27451436

[17] 17. Dávila-Cervantes C, Agudelo-Botero M. Changes in life expectancy due to avoidable and non-avoidable deaths in Argentina, Chile, Colombia and Mexico, 2000–2011. Cad Saude Publica. 2018 Jun;34(6):e00093417. doi: 10.1590/0102-311X00093417 10.1590/0102-311X00093417 29947656

[18] 18. Frenk J. Leading the way towards universal health coverage: a call to action. Lancet. 2015 Apr;385(9975):1352–8. doi: 10.1016/S0140-6736(14)61467-7 10.1016/S0140-6736(14)61467-7 25458718

[19] 19. Borges GM. Health transition in Brazil: regional variations and divergence/convergence in mortality. Cad Saude Publica. 2017 Aug;33(8):e00080316. doi: 10.1590/0102-311X00080316 10.1590/0102-311X00080316 28832781

[20] 20. Schramm JMDA, Oliveira AFD, Leite IDC, Valente JG, Gadelha ÂMJ, Portela MC, et al. Transição epidemiológica e o estudo de carga de doença no Brasil. Cien Saude Colet. 2004;9(4):897–908.

[21] 21. Szot Meza J. La transición demográfico-epidemiológica en Chile, 1960-2001. Rev Esp Salud Publica. 2003;77:605-13. 14608963

[22] 22. Valdivia G. Transición epidemiológica: la otra cara de la moneda. Rev Med Chil. 2006 Jun;134(6):675-8. 10.4067/s0034-98872006000600001 17130940

[23] 23. Mayorga C. Tendencia de la mortalidad y sus determinantes como parte de la transición epidemiológica en Colombia. Gerenc Polit Salud. 2004 Dec;3(7):62-76.

[24] 24. Martinez CS, Leal GF. Epidemiological transition: model or illusion? A look at the problem of health in Mexico. Soc Sci Med. 2003 Aug;57(3):539-50. 10.1016/s0277-9536(02)00379-9 12791495

[25] 25. Huynen MM, Vollebregt L, Martens P, Benavides BM. The epidemiologic transition in Peru. Rev Panam Salud Publica. 2005;17(1):51-9. 10.1590/s1020-49892005000100010 15720881

[26] 26. Huarcaya WV, Miranda J, Ramos W. Situación de la transición epidemiológica a nivel nacional y regional: Perú, 1990-2006. Rev Peru Epidemiol. 2011 Dec;15(3):2-5.

[27] 27. World Health Organization. Global Health Observatory (GHO) data. Available from: https://www.who.int/gho/countries/en/ Accessed on 15 December 2018.

[28] 28. World Health Organization. Health statistics and information systems. Available from: https://www.who.int/healthinfo/statistics/mortality_rawdata/en/ Accessed on 15 December 2018.

[29] 29. United Nations, Department of Economic and Social Affairs, Population Division. World population prospects 2019: highlights. New York: UN; 2019. Available from: https://population.un.org/wpp/Publications/Files/WPP2019_Highlights.pdf Accessed on 20 August 2019.

[30] 30. Hoem JM. Life table. In: Wright JD, ed. International encyclopedia of the social & behavioral sciences. Second ed, vol. 14. Amsterdam: Elsevier; 2015:89-92.

[31] 31. Weerasinghe DP, Parr NJ, Yusuf F. Analysis using life tables of the differences between country of birth groups in New South Wales, Australia. Public Health. 2009 May;123(5):351-7. 10.1016/j.puhe.2009.03.006 19443003

[32] 32. de Andrade LOM, Pellegrini Filho A, Solar O, Rígoli F, de Salazar LM, Serrate PCF, et al. Social determinants of health, universal health coverage, and sustainable development: case studies from Latin American countries. Lancet. 2015 Apr;385(9975):1343-51. doi: 10.1016/S0140-6736(14)61494-X 10.1016/S0140-6736(14)61494-X 25458716

[33] 33. Atun R, de Andrade LOM, Almeida G, Cotlear D, Dmytraczenko T, Frenz P, et al. Health-system reform and universal health coverage in Latin America. Lancet. 2015 Mar/Apr;385(9974):1230-47. doi: 10.1016/S0140-6736(14)61646-9 10.1016/S0140-6736(14)61646-9 25458725

[34] 34. Saad PM. Demographic trends in Latin America and the Caribbean. In: Cotlear D, ed. Population aging: Is Latin America ready? Washington, D.C.: World Bank; 2011:43-77.

[35] 35. Schraiber LB, D’oliveira AFPL, Couto MT. Violência e saúde: estudos científicos recentes. Rev Saude Publica. 2006;40:112-20. 10.1590/s0034-89102006000400016 16924311

